# Identification of the Sex-Biased Gene Expression and Putative Sex-Associated Genes in *Eucommia ulmoides* Oliver Using Comparative Transcriptome Analyses

**DOI:** 10.3390/molecules22122255

**Published:** 2017-12-18

**Authors:** Wencai Wang, Xianzhi Zhang

**Affiliations:** 1 Institute of Clinical Pharmacology, Guangzhou University of Chinese Medicine, Guangzhou 510000, China; wencaiwang@gzucm.edu.cn; 2 Department of Forestry Protection, College of Forestry, Northwest A&F University, Yangling 712100, China

**Keywords:** *Eucommia ulmoides*, dioecious plant, sexual dimorphism, sex-associated genes, transcriptome sequencing

## Abstract

*Eucommia ulmoides* is a model representative of the dioecious plants with sex differentiation at initiation. Nevertheless, the genetic mechanisms of sexual dimorphism and sex determination in *E. ulmoides* remain poorly understood. In this study *de novo* transcriptome sequencing on Illumina platform generated >45 billion high-quality bases from fresh leaves of six male and female individuals of *E. ulmoides*. A total of 148,595 unigenes with an average length of 801 base-pairs (bp) were assembled. Through comparative transcriptome analyses, 116 differentially expressed genes (DEGs) between the males and the females were detected, including 73 male-biased genes and 43 female-biased genes. Of these DEGs, three female-biased genes were annotated to be related with the sexually dimorphic gutta content in *E. ulmoides*. One male-biased DEG was identified as putative MADS box gene APETALA3, a B class floral organ identity gene in the flowering plants. SNPs calling analyses further confirmed that the APETALA3-like gene was probably involved in the sex determination in *E. ulmoides*. Four other male-biased DEGs were potential sex-associated genes as well with segregated SNPs in accord with sex type. In addition, the SNPs density was 1.02 per kilobase (kb) in the expressed genes of *E. ulmoides*, implying a relatively high genetic diversity.

## 1. Introduction

*Eucommia ulmoides* Oliver is a dioecious tree species of the family Eucommiaceae, in the asterid lineage of the angiosperms [1], that is endemic to southern and central China [2]. It is a Tertiary relict species that has been a well-known medicinal plant in China for >2000 years [3]. Recently, the high productivity of gutta, i.e., *trans*-1,4-polyisoprene (TPI) in *E. ulmoides* has attracted broad attention [4,5,6]. *E. ulmoides* therefore has the potential to replace the commonly known *para*-rubber tree (*Hevea brasiliensis* from the Euphorbiaceae, which only grows in tropical zones) because of its wide distribution in the subtropical and temperate ecozones and good resistance [7,8]. However, sex-identification of the young seedlings of *E. ulmoides* is difficult due to its dioecious sexual system and long-life cycle, which constrains its breeding process [9]. Moreover, the yields of gutta in the leaves differ significantly between male and female *E. ulmoides* trees [10], making it thus essential to identify the sex-associated genes for molecular sex-typing of *E. ulmoides* to assist the breeding progress for improving the yield of gutta or other target traits.

About 6% flowering plants (15,600 species) are dioecious and dispersed in 987 genera belonging to 175 families [11]. Among these only 39 species from 15 families are confirmed by reliable cytogenetic and/or molecular evidence to have sex chromosomes [12]. As a dioecious diploid species (2*n* = 34, 1C DNA value per haploid genome = 723.7 megabases (Mb)) [13,14], whether *E. ulmoides* has a heteromorphic sex chromosome(s) or not is still uncertain [10,15]. Nevertheless, the strict dioecy and nearly 1:1 sex ratio of *E. ulmoides* in Nature both suggest that the sex of this species is most likely controlled by a genetic mechanism [10,15,16]. Due to the inhibited recombination of sex determination regions it is hard to identify the sex determination genes by classical approaches [17,18,19]. The advances of next generation sequencing (NGS) technique have broadly facilitated sex determination studies in flowering plants (reviewed in [20]). NGS-based transcriptome sequencing has widely been used in the identification of the differentially expressed genes (DEGs) associated with sex differentiation in cucumber (*Cucumis sativus*) [21], white campion (*Silene latifolia*) [22], *Rumex hastatulus* [23], and garden asparagus (*Asparagus officinalis*) [24]. Nevertheless, to our knowledge the sex determination mechanisms and sex-related DEGs in *E. ulmoides* remain unknown to date.

Historically two types of unisexual flowers have been recognized, i.e., type I and type II [25,26]. The first type unisexual flowers (type I) are initially bisexual and subsequently develop into unisexual ones by abortion of the androecium (in female flowers) or gynoecium (in male flowers), whereas the second type of unisexual flowers (type II) only initiate development of one reproductive organ resulting in either female or male flowers [26]. It has been revealed that in *E. ulmoides* there is no occurrence of stamen primordia in the female trees neither is there pistil primordia in the male trees [27], causing the “naked” flowers i.e., achlamydeous flowers with only either a pistil or stamen. It is thus rational to assume that the unisexual flowers in *E. ulmoides* belong to type II, like in its closely-related taxa *Aucuba chinensis* and *Garrya elliptica* of the family Garryaceae [28,29].

In the ABC model of flower development, the B and C class organ identity genes are responsible for determining stamen and carpel initiations [30]. The genes APETALA3 (AP3), PISTILATA (PI), AGAMOUS (AG) have been identified as key members of the ABC model in *Arabidopsis* with the former two as B class genes whereas the latter one as C class gene [31]. All the B and C class genes identified in the flowering plants including the three above genes belong to the MADS box TF (transcription factor) family and are probable sex-associated genes segregating with gender [32,33,34]. It has been demonstrated that the B class genes are essential for the identity of stamens at initiation in the dioecious plants *Spinacia oleracea* [35] and *Thalictrum dioicum* [36]. We thus speculate here that MADS box genes probably contribute to the sex differentiation of *E. ulmoides* and probably express constitutively, in line with the hypothesis proposed by Diggle et al. [26] stating that for the dioecious species with type II unisexual flowers the sex determining genes are limited among the genes in the pathway from floral commitment to floral organ identity. 

Sexual dimorphism such as differences in the morphology and physiology is commonly present in the dioecious plants, which may be related to the DEGs [37,38] or other genetic and epigenetic factors between the male and female individuals. In plants, isopentenyl diphosphate (IPP) is the precursor of polyisoprene, which is synthesized in two distinct pathways, i.e., the methylerythritol phosphate (MEP) pathway in the plastids and the mevalonate (MVA) pathway in the cytoplasm [6,39]. Gutta i.e., TPI, is formed by successive condensation of the IPP into a *trans*-structure [5]. Pyruvate is a necessary raw material in the MEP pathway reacting with glyceraldehyde 3-phosphate (DXP) to produce 1-deoxy-D-xylulose 5-phosphate that is the primary substrate for the synthesis of IPP [5,40]. Since gutta yields differ in the leaves of the male and female *E. ulmoides* [10] we therefore hypothesize that the genes in the gutta synthesis pathways are likely expressed at different levels and further contribute to the sexual dimorphism of gutta content in the female and male leaves of *E. ulmoides*, which is essential to further detect. In the present study, large-scale NGS transcriptome data were generated from the fresh leaves of the male and female samples in three independent full-sib families of *E. ulmoides* by RNA-seq method in combination with comprehensive comparative analyses. Through these investigations we aimed to: 1) detect the DEGs related to the sexual dimorphism of gutta yield in the male and female leaves of this economically important tree species; and 2) identify potential sex-associated genes in *E. ulmoides*.

## 2. Results

### 2.1. NGS Sequencing and De Novo Transcriptome Assembly

A total of six individuals of *E. ulmoides* from three independent full-sib families with each sex having three individuals were sequenced according to Illumina PE (paired-end) sequencing protocol. About eight to ten gig abases (Gb) raw sequencing data were generated for each individual, reaching to a total of 327,129,846 raw reads in all the samples. After all the sequenced reads were quality-filtered by removing reads containing adapter sequences, ambiguous nucleotides, low-quality sequences, and all possible contaminations, c. eight Gb clean data were obtained for each individual. A total number of 311,326,872 clean reads (46.71 Gb) were eventually retained, of which 161,269,666 were from the male trees and 150,057,206 were from the female trees (Table 1). The low sequencing error rate (0.02%) and high Q20 (>95%) and Q30 (>89%) values were all indicative of high-quality data. The average GC-nucleotide content of samples was 41.96%. 

To improve the quality and integrality of the assembly, we combined the sequencing data of six libraries for de novo transcriptome assembly. A total of 289,704 contigs with mean length of 540 base-pairs (bp) were assembled. The N50 contig size, the minimum and maximum contig length were 705, 201, and 12,536 bp, respectively. These contigs were further assembled into 148,595 unigenes with an N50 length of 1064 bp and average length of 801 bp. The transcript with the longest length of each unigene was selected as the reference sequence for further analysis. The total length of all the unigenes reached to 117,298,413 bp (Table 2). Furthermore, more than half (82,321%, 55.4%) of the unigenes were longer than 500 bp and c. a quarter (34,178, 23.0%) of the unigenes were longer than 1000 bp. The transcriptome data have been deposited in the Sequence Read Archive (SRA) with the accession number of PRJNA399774. 

### 2.2. Transcriptome Annotation and Functional Classification

All the assembled unigenes were validated and annotated by blatsx searching against seven public databases i.e., NCBI non-redundant protein sequences (Nr) and NCBI non-redundant nucleotide sequences (Nt) on the website of www.ncbi.nlm.nih.gov; Protein family (Pfam, http://xfam.org/); euKaryotic Ortholog Groups (KOG, www.ncbi.nlm.nih.gov/COG); Swiss-Prot (a manually annotated and reviewed protein sequence database, www.expasy.ch/sprot); KEGG Ortholog database (KO, www.genome.jp/kegg); and Gene Ontology (GO, http://www.geneontology.org/). Among the total 148,595 unigenes, 84,271 (56.71%) unigenes were annotated successfully in at least one database (Table S1). There were 71,810 (48.32%), 49,521 (33.32%), 24,028 (16.17%), 55,473 (37.33%) and 54,515 (36.68%) unigenes having homologous sequences in the databases of Nr, Nt, KOG, GO and Pfam, respectively (Table S1).

**Figure 1:**
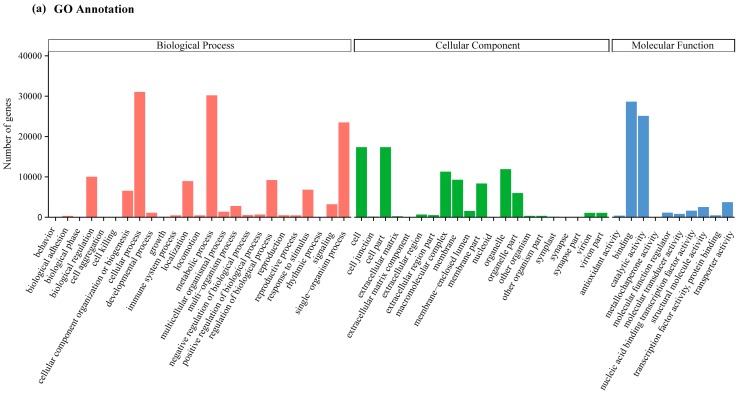

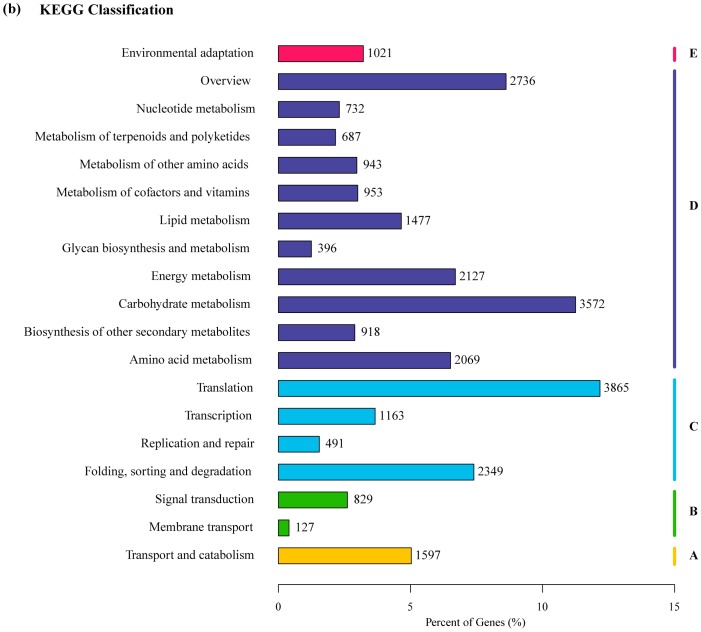
Transcriptome annotation of *Eucommia ulmoides* leaves. (**a**) Gene Ontology (GO) annotation of non-redundant consensus sequences of *Eucommia ulmoides*. Most consensus sequences against GO database were grouped into three major functional categories i.e., biological process (BP), cellular component (CC), and molecular function (MF); (**b**) Pathway assignment of *Eucommia ulmoides* unigenes based on the Kyoto Encyclopedia of Genes and Genomes (KEGG) database. (**A**) Cellular process categories; (**B**) environmental information processing categories; (**C**) genetic information processing categories; (**D**) metabolism categories; and (**E**) organismal systems categories.

Furthermore, the unigene sequences were characterized by assigning to the GO terms (Figure 1a). In total, 2222 functional GO terms were eventually assigned. The most highly represented categogries of biological process were cellular processes (31,057 unigenes), metabolic processes (30,211 unigenes), and single-organism processes (23,497 unigenes). Similarly, under the classification of cellular component, cell (17,355) and cell part (17,352) were the two mostly represented. For the categories of molecular functions, the binding (28,622 unigenes) and catalytic activities (25,112) represented the two largest categories. In addition, the 24,028 unigenes that were aligned to the KOG classification of 26 categories were further analyzed using the KEGG pathway database. Out of the total 148,595 unigenes assembled, 31,746 (21.36%) were assigned to 131 KEGG pathways belonging to 19 classifications in five main categories (Figure 1b). The top 10 categories assigned in the KEGG database were belonged to translation (3865, 12.2%), carbohydrate metabolism (3572, 11.3%), overview (2736, 8.6%), folding, sorting and degradation (2349, 7.4%), energy metabolism (2127, 6.7%), amino acid metabolism (2069, 6.5%), transport and catabolism (1597, 5.0%), lipid metabolism (1477, 4.7%), transcription (1163, 3.7%), and environmental adaptation (1.021, 3.2%) (Figure 1b).

### 2.3. Transcriptomes Comparison between the Males and the Females

**Figure 2:**
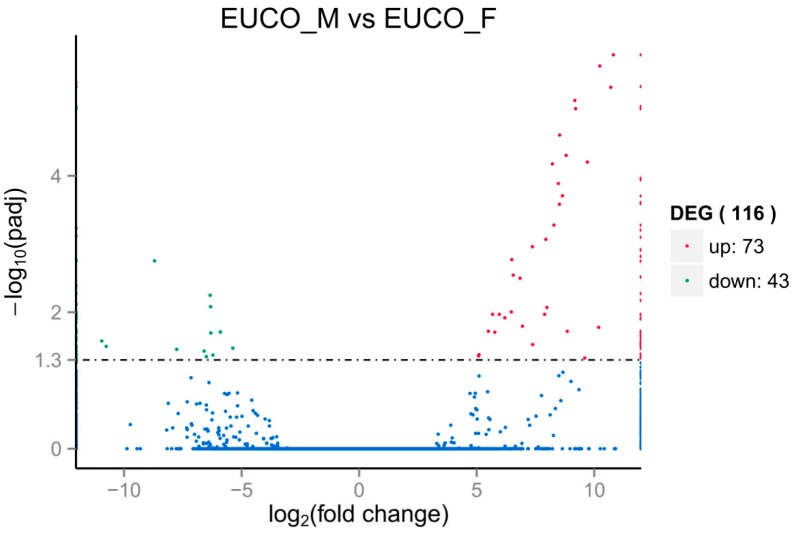
Volcano plot of the identification of DEGs (differentially expressed genes) between the males and the females of *Eucommia ulmoides*. *X* axis represents the log_2_ (fold change value), while *Y* axis represents –log_10_ (*p*_adj_ value). Green dots show the female-biased genes, and red dots represent the male-biased genes. EUCO_M: Males; EUCO_F: Females.

To reveal the sex-biased gene expression, the clean reads of the male and female samples were each grouped into one dataset respectively for digital expression comparative analysis. Using the criteria of an adjusted *p*-value (*p*_adj_) < 0.05 and a minimal twofold difference in expression i.e., |log_2_ (fold change value)| ≥ 1, we found that 116 genes were differentially expressed in the male and female leaves, including 73 (62.9%) male-biased (up-regulated) and 43 (37.1%) female-biased (down-regulated) genes (Table S2, Figure 2). All these genes were strongly up- (log_2_ (fold change value) ≥ 5) or down- (log_2_ (fold change value) ≤ −5) regulated. Among these DEGs, sex-specific genes that expressed in either males or females were further investigated. We used the following criteria: DEGs had an estimated abundance of zero read counts in one sex but contained a certain expression in the other sex i.e., fragment per kilobase per million (FPKM) value >0.03. The result showed that 71 DEGs were identified as sex-specific genes, including 40 male-specific genes and 31 female-specific genes (Table S2).

**Figure 3:**
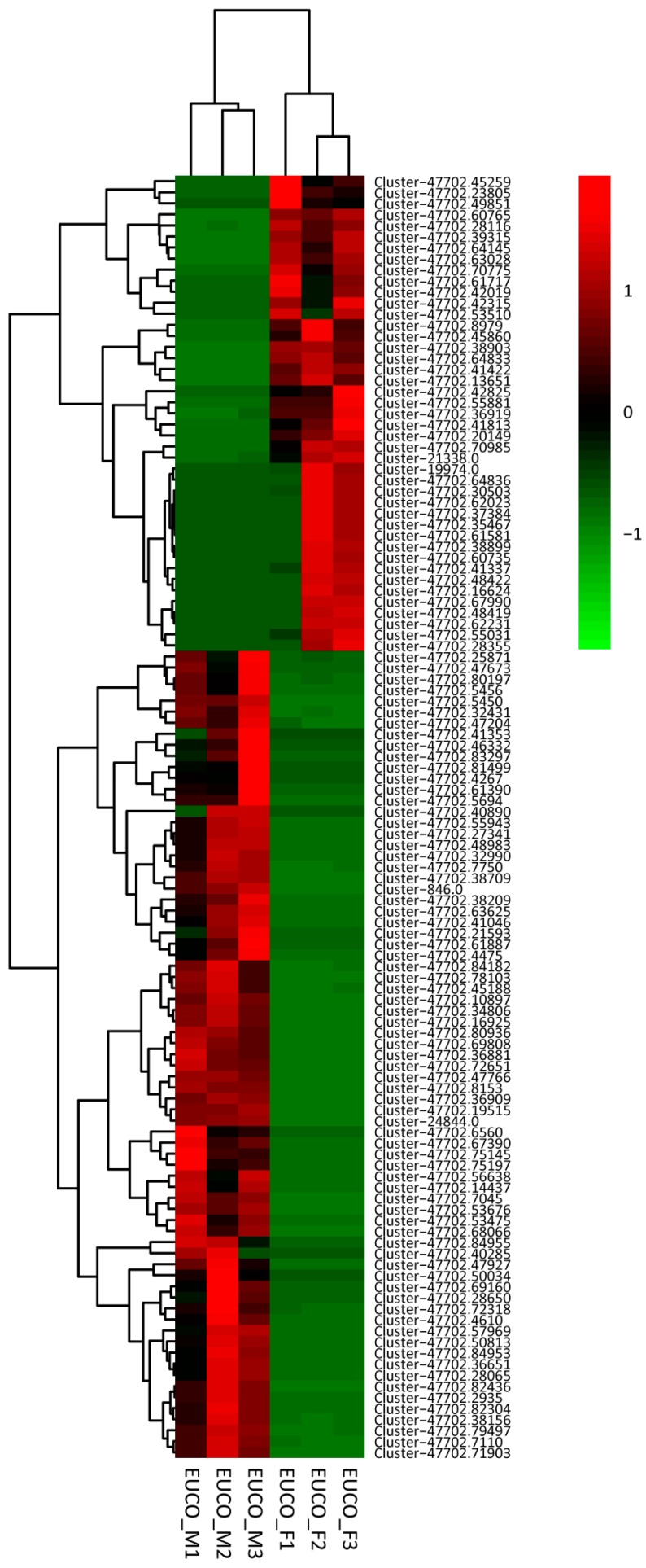
Heat map diagram of expression patterns for 116 DEGs (differentially expressed genes) between the males and the females of *Eucommia ulmoides*. The color from red to green indicates the gene expression level towards small. EUCO_M: Males, EUCO_F: Females; the numbers 1/2/3 represent different individuals.

The expression patterns of the determined 116 DEGs among the six male and female individuals of *E. ulmoides* were further analyzed by K-means clustering method using the pheatmap R package. The result revealed that the six individuals were clustered according to sex types i.e., males in a clade and females in another one (Figure 3). Seventy-three genes were highly expressed in three males while 43 genes had female-biased expression (Figure 3), which confirmed the result of DESeq analysis (Figure 2). Moreover, genes with similar expression level clustered together, which may also show similar functions. For example, genes Cluster-47702.13651 and Cluster-47702.41422 located in a clade and both involved in the malate metabolism (Table S2, Figure 3).

**Figure 4:**
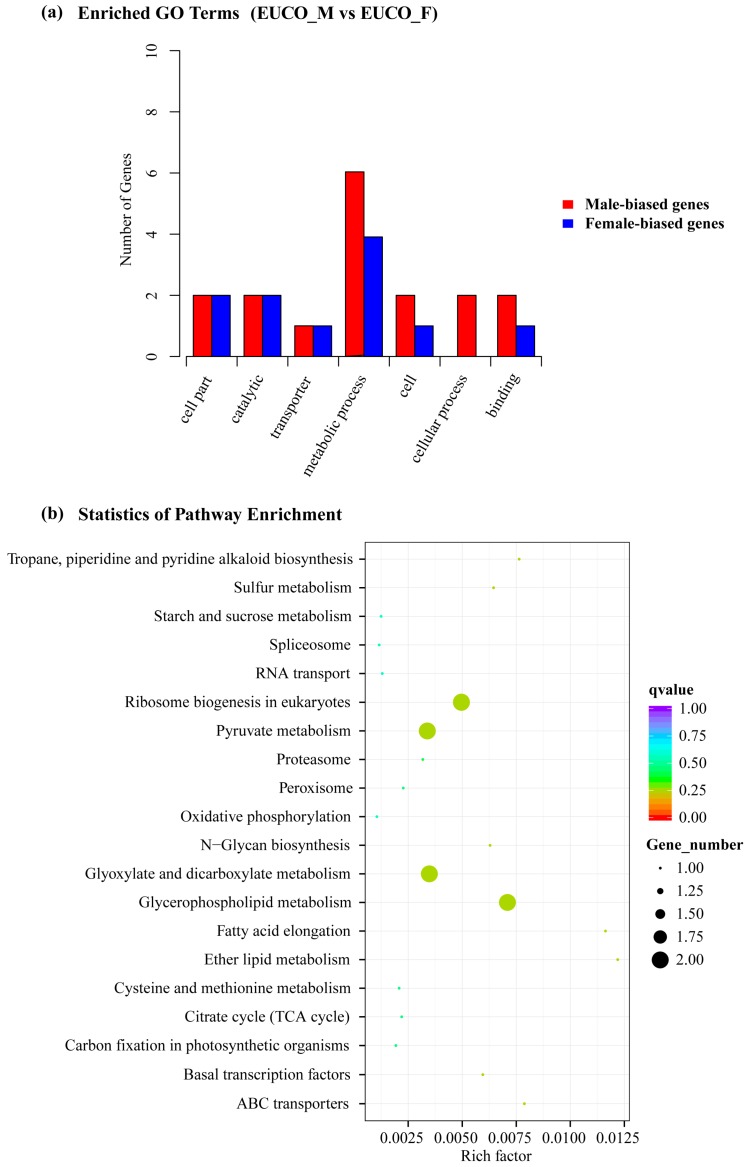
Gene Ontology (GO) and Kyoto Encyclopedia of Genes and Genomes (KEGG) enrichments of 116 DEGs (differentially expressed genes) between the males and the females of *Eucommia ulmoides*. (**a**) Statistics of GO terms enrichment of the DEGs. The *Y*-axis represents the number of DEGs in a category. EUCO_M: Males; EUCO_F: Females; (**b**) Statistics of KEGG pathway enrichment of the DEGs. The *Y* axis represents the different KEGG pathways, and the *X* axis shows the rich factor. The color scale indicates the *q*-value range, and the size of the circles means the gene numbers.

GO enrichment analysis was conducted by GOseq using Wallenius non-central hyper-geometric distribution to further characterize the DEGs between the males and the females of *E. ulmoides*. We found that 28 male- and female-biased genes were enriched in seven GO terms, namely cell part, catalytic activity, transporter, metabolic process, cell, cellular process, and binding (Figure 4a), suggesting obviously sexual dimorphism in terms of basic metabolic activity in the two sex types of *E. ulmoides*. Among these genes, one female-biased gene (Cluster-47702.13651) having homology with the MLS gene (malate synthase) of *Solanum* was enriched in pyruvate metabolic process (GO:0006090) (Table S2). Similarly, one male-biased gene (Cluster-47702.28065) enriched in malate metabolic process (GO:0019643) was homologous to the MDH gene (malate dehydrogenase) of *Solanum* (Table S2). Another female-specific expressed gene (Cluster-47702.70775) showed some similarity with the TPP gene (trehalose-6-phosphate phosphatase) of *Arabidopsis*, likely involved in glycometabolism (Table S2). In addition, we found that one female-specific gene (Cluster-47702.60735) was enriched in the GO term of gene silencing by RNA (GO:0031047) (Table S2). This gene had homology with the RDM1 gene (RNA-directed DNA methylation 1) of *Arabidopsis*, involved in the RdDM (RNA-directed DNA methylation) epigenetic pathway (Table S2). There were several othergenes e.g., Cluster-47702.39315 and Cluster-47702.36919 involved in photosynthesis (GO:0015979) and mitochondrial proton-transporting ATP synthase (GO:0000276).

To further determine whether the sex-biased genes were involved in specific pathways related to gutta biosynthesis, we performed KEGG enrichment analysis using KOBAS. The result revealed that a total of 25 DEGs were assigned to 21 apparently enriched KEGG biochemical pathways from the index of rich factor (number of DEGs/total number of unigenes annotated in the same pathway), q-value and gene number (Table S2). The top 20 significantly enriched pathways, shown in Figure 4b, reveal that the “pyruvate metabolism”, “glyoxylate and dicarboxylate metabolism”, “glycerophospholipid metabolism”, and “ribosome biogenesis in eukaryotes” pathways enriched the most DEGs, each with two sex-biased genes. Two genes (MLS and MDH) that participated in the pyruvate metabolism (ko00620) were differently expressed between the male and female leaves of *E. ulmoides* (Table S2, Figure S1). These two genes also played a part in “glyoxylate and dicarboxylate metabolism” (ko00630) (Table S2). For “glycerophospholipid metabolism” (ko00564), NMT1 (Cluster-47702.45259, phosphoethanolamine *N*-methyltransferase) was highly expressed in females, while LPCAT1_2 (Cluster-47702.68066, lysophosphatidylcholine acyltransferase) was highly expressed in males. For “starch and sucrose metabolism” (ko00500), the expression of TPP gene was only detected in females (Table S2). Two male-biased genes i.e., MPP10 (Cluster-47702.7045) and MDN1 (Cluster-47702.79497) were assigned to “ribosome biogenesis in eukaryotes” (ko03008) by KEGG (Table S2). One male-specific gene NDUFV1 (Cluster-47702.41353) was assigned to “oxidative phosphorylation” (ko00190). The above enriched GO terms and KEGG pathways (Figure 4) may to some degree account for the difference of gutta content in the female and male leaves of *E. ulmoides*.

### 2.4. Differential Expression of MADS Box Genes in the Males and the Females

**Figure 5:**
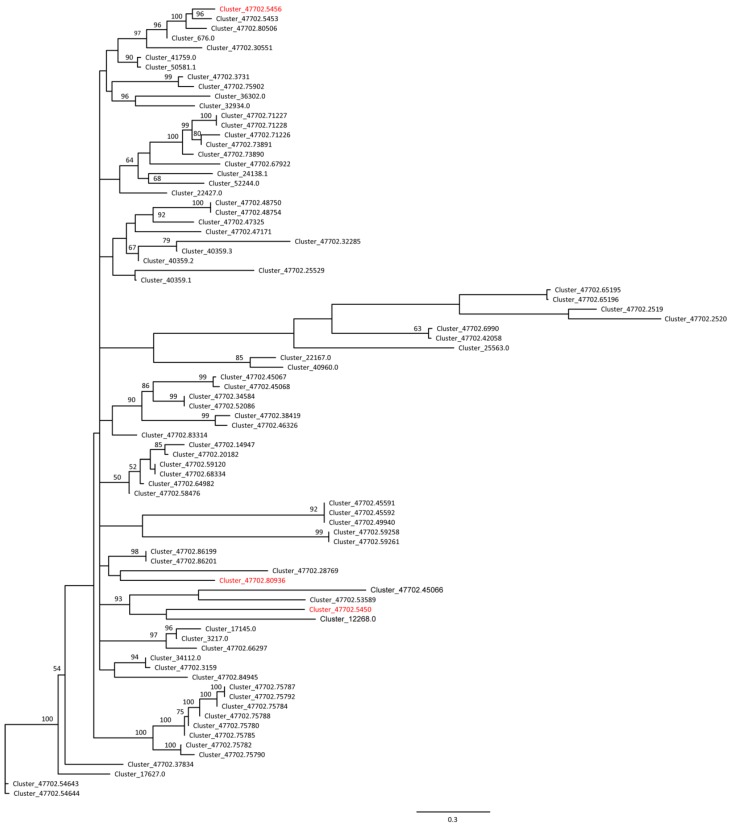
The NJ (neighbor joining) tree of putative 100 MADS box TF (transcription factor) genes in *Eucommia ulmoides*. The differentially expressed MADS genes between the males and females of *E. ulmoides* are indicated in red. Support values >50% were shown above the nodes.

The search of MADS box TFs in the assembled transcriptome of *E. ulmoides* identified 100 putative MADS box unigenes (Table S3). Of these unigenes, three (Cluster-47702.80936, Cluster-47702.5456 and Cluster-47702.5450) were differentially expressed between male and female leaves (Figure 2 and Figure 3, Table S2). Further detection revealed that all the three MADS box genes were preferentially expressed in males, which could stochastically be caused by the finding of more male-biased genes. One male-biased MADS box TF gene with length of 1345 bp (Cluster-47702.80936) was homologous to the *Arabidopsis* AP3 gene, a B class organ identity gene, with high similarity (Table S2 and Table S3). The other two MADS box TF genes were homologous to the AGAMOUS-like 8 (AGL8) gene of *Glycine* or *Solanum*, a C class organ identity gene, although with relatively low similarity (Table S2). In addition, the neighbor joining (NJ) tree of the putative 100 MADS box unigenes of *E. ulmoides* showed that the above three genes were separately located in different clades, all of which werehighly supported (>90%) (Figure 5).

**Figure 6:**
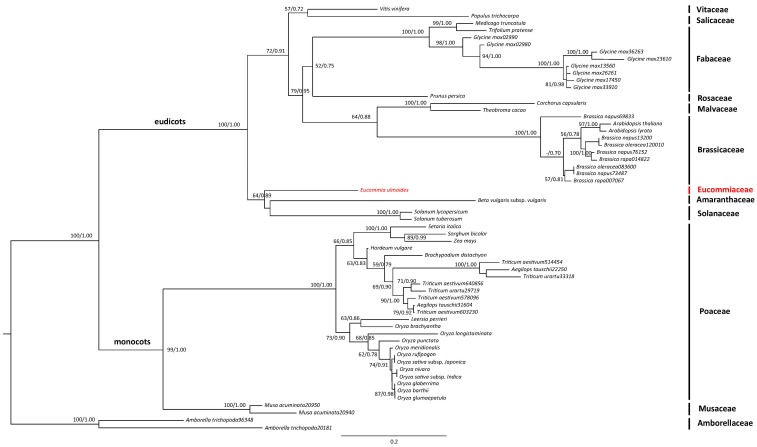
The maximum-likelihood phylogenetic tree constructed using 58 AP3 orthologous sequences in 38 angiosperms. *Eucommia ulmoides* are colored in red, and the numbers above the nodes represent the support values (MLBP/PP). The numbers after the species name indicate different gene copies.

The maximum likelihood (ML) and Bayesian inference (BI) analyses based on 58 AP3 orthologues from 38 angiosperms produced a congruent tree as shown in Figure 6. Two clades were revealed with high support values, corresponding to monocots (99/1.00) and eudicots (100/1.00). All the sampled families were highly supported as monophyly, e.g., Brassicaceae (100/1.00), Fabaceae (100/1.00) and Poaceae (100/1.00). *E. ulmoides* (Eucommiaceae) was revealed to be located in eudicots correlating to species from Solanaceae and Amaranthaceae with low support values (64/0.89). Brassicaceae, Fabaceae, Malvaceae and Rosaceae clustered together in a clade with medium level supports (79/0.95), which then sistered to a clade comprising of Salicaceae and Vitaceae (72/0.91). However, the relationships of species from large families like Poaceae and Brassicaceae, were not well resolved here.

### 2.5. SNPs Detection in the DEGs and the Whole Transcriptomes

The detection of SNPs (single nucleotide polymorphisms) occurrence in the DEGs revealed that a total of 24 DEGs were polymorphic in *E. ulmoides*, including 16 male-biased genes and eight female-biased genes (Table S4). Further examination of polymorphic DEGs with common SNPs in each sex type identified five potential sex-associated genes, i.e., Cluster-47702.80936, Cluster-47702.80197, Cluster-47702.38156, Cluster-47702.79497 and Cluster-47702.45188 (Table 3). There was one base substitution event in the 1101th site of Cluster-47702.80936 (from the direction of 5′ to 3′), with nucleotide C fixed in the females and G in the males. The other four genes (Cluster-47702.80197, Cluster-47702.38156, Cluster-47702.79497 and Cluster-47702.45188) fixed SNPs as T↔C, G↔A, T↔C, G↔T, A↔G, C↔T, T↔G, and T↔C in the females and the males, respectively (Table 3). The five putative sex-associated DEGs all had male-biased expression patterns. It is noteworthy that the polymorphic male-biased DEG Cluster-47702.80936 was a MADS box TF gene and homologous to the B class organ identity gene AP3 of *Arabidopsis*, *S. oleracea* and other flowering plants (Figure 6 and Table S2). Nevertheless, SNPs that separated with sex types were not detected in the other two MADS box genes i.e., Cluster-47702.5456 and Cluster-47702.5450 (Tables S2 and S4).

The SNPs calling analyses from the six transcriptomes revealed that the genetic variance in *E. ulmoides* genes was plentiful (Table 4 and Table S5) with a total of 78,563 nucleotide transitions and 40,848 nucleotide transversions (Table 4). The average frequency of SNPs occurrence in the expressed genes of *E. ulmoides* was 1.02 per kilobase (kb), amounting to a total of 119,411 SNPs (Table 4). Further examination of SNPs in the CDS (coding sequence) regions suggested that most of nucleotide substitutions occurred in the first (22,235) and third (22,148) position of codons. The amount of SNPs varied from 32,570 in one male (EUCO_M1) to 57,340 in another male (EUCO_M3) (Table S5) among six individuals. In all the examined male and female samples there were more than half SNPs occurred in the non-coding regions rather than in the coding areas. More synonymous SNPs than nonsynonymous SNPs were detected in each individual (Table S5).

## 3. Discussion

### 3.1. Genes Related to Sexual Dimorphism of Gutta Content in the Leaves of E. ulmoides

Plant sexual dimorphisms are widespread although the females and males are often genetically similar [37,38]. Genes that are primarily expressed in one sex over the other, i.e., genes with sex-biased expression, often contribute largely to the expression of sexually dimorphic traits [41,42,43]. The leaves of *E. ulmoides* are sexually dimorphic regarding the gutta content [10]. In this study, 148,595 unigenes derived from the fresh healthy leaf tissues of both the male and female individuals of *E. ulmoides* were assembled and analyzed, of which 57% were well annotated in the public databases. Through comparative transcriptome analyses a total of 116 DEGs were detected in the male and female leaves, of which 73 and 43 were overexpressed or specifically expressed in the males and females respectively (Figure 2 and Figure 3, Table S2). Subsequent functional enrichment analyses of the DEGs revealed various sex-dimorphic physiological processes, such as the pyruvate synthesis, starch and sucrose metabolism, and DNA methylation pathway in two sexes of *E. ulmoides* (Figure 4).

Two distinct pathways i.e., the MEP pathway and the MVA pathway play key roles in the synthesis of IPP which is the precursor of polyisoprene [6,39]. Pyruvate is a primary material in the MEP pathway [5,40]. Our comparative analysis here revealed that the MLS gene was specifically expressed in *E. ulmoides* female leaves and enriched in the pyruvate metabolism pathway (Figure 4 and Table S2). Malate can then be transformed into pyruvate via the intermidate oxaloacetate (Figure S1, ko00620, www.genome.jp/kegg). It is likely that the female-specific expression of MLS lead to higher accumulation of malate, which produced more pyruvate, and finally higher content of gutta is yielded [10]. It may also imply that gutta in the leaves might be synthesized using IPP molecules produced from the MEP pathway, as revealed in the stems of *E. ulmoides* [5]. It is noteworthy that the MDH gene that catalyzed the transformation between the oxaloacetate and the malate (Figure S1, ko00620, www.genome.jp/kegg) was preferentially expressed in the male leaves. This is suggestive of a fine dynamic regulation process of pyruvate concentration in the *E. ulmoides* leaves. Furthermore, Acetyl-CoA produced through the glycolysis/gluconeogenesis metabolism is a precursor of mevalonic acid in the MVA pathway [40,44]. Here we found that TPP, one female-specific expressed gene was enriched in the starch and sucrose metabolism (Figure 4 and Table S2). The significantly higher expression of TPP in the female leaves of *E. ulmoides* could induce a higher concentration of glucose (ko00500, www.genome.jp/kegg), which likely further regulates the downstream metabolisms to accumulate more gutta [10]. This is similar as in *H. brasiliensis* where the efficiency of sugar metabolism seemed to be positively associated with the rubber yield [45]. 

Interestingly, we found one female-specific gene (2405 bp) that is homologous to the gene RDM1 in *Arabidopsis* (Table S2). RDM1 is reported to be an indispensable factor for the RNA polymerase V (Pol V) function in the canonical RdDM pathway, a critical epigenetic mechanism to silence the protein-coding genes and DNA repeats (e.g., LTR retrotransposons) [46], in the angiosperms [47,48]. The finding of sex-biased expression of RDM1 homologue in *E. ulmoides* indicates that regulations at the epigenetic level probably play an important role in shaping the sexual dimorphisms. In addition, a number of DEGs, e.g., NMT1, NDUFV1and MDN1, were shown to be enriched in several other basic metabolic processes, for instance “glyoxylate and dicarboxylate metabolism”, “ribosome biogenesis in eukaryotes”, ”glycerophospholipid metabolism” and “carbon fixation in photo-synthesis” (Figure 4). The difference in these physiological progresses between the two sexes of *E. ulmoides* may also take part in the sexual dimorphism of gutta content. Moreover, these distinct processes are probably indicating that sexual divergences of *E. ulmoides* could be present in the aspects of respiratory metabolism, protein synthesis, and photosynthesis rate as well. Further substantial studies are needed to comprehensively understand the sexual dimorphisms in *E. ulmoides* in the future.

### 3.2. A MADS Box TF Gene Involved in Sex Differentiation of E. ulmoides

The sex determination of plant has long been an intriguing and intractable issue since Darwin’s time [25]. Sex determination genes in dioecious species with type II unisexual flowers such as in *E. ulmoides* [27] are the genes occurred from the floral initiation to the reproductive organ formation, particularly the B and/or C class floral identity genes [26]. In angiosperms c. 42 genera including *Aucuba* [28], *Eucommia* [27] and *Garrya* [29] in the Garryales clade in asterids have type II unisexual flowers. As the only living species of the family Eucommiaceae, *E. ulmoides* has diverged from its close lineages for >48 million years [49,50], indicating a long period of evolution of the type II unisexual flowers.

TF genes have been previously suggested to control the plant gender differentiation, for example, the CmWIP1 gene in cucumber [51] and the MeGI gene in persimmon [52]. MADS box TF family including all the recognized B (e.g., AP3 and PI) and C (e.g., AG) class genes is of particular importance in the sex determination of dioecious plants [26,53,54,55,56]. One MADS box TF gene controlling the identity of stamen primordia was found to be DEG in the male and female flowers of kiwifruit (*Actinidia chinensis*) [57]. One MADS box gene in the shrub willow (*Salix suchowensis*) was recorded to express significantly higher in the female flower buds than in the males [58]. Several MADS box TF genes in the garden asparagus (*A. officinalis*) also expressed differentially in the two sex types of flower buds [59]. In this study, 100 putative MASD box unigenes were identified from the *E. ulmoides* transcriptome data (Table S3), this number is similar as the MADS box genes (112) found in tomato (*Solanum lycopersicum*) [60]. We found that three out of the 100 MADS box TF genes were DEGs between the males and females *E. ulmoides* (Figure 2 and Figure 5, Table S2). One of them (Cluster-47702.80936) had homology with the B class gene AP3 in *Arabidopsis* with high similarities (Table S2). Further ML and BI phylogenetic analyses of the AP3-like gene from *E. ulmoides* and 57 AP3 orthologues from other 37 flowering plants produced a congruent tree (Figure 6). Monocots and eudicots were both highly supported, as well as for the monophyly of every sampled family including Brassicaceae, Fabaceae, Malvaceae, Poaceae and Solanaceae, which agreed with the largely accepted phylogeny of angiosperms at present [1,61]. This further confirmed the homology of AP3-like genes in *E. ulmoides* with the AP3 genes in other flowering plants.

The B and C class MADS box genes control the initiation of stamens and carpels [33,34]. For instance, in *Aquilegia*, homologs of B class genes AP3 and PI are necessary for stamen identity [62]. In *T. dioicum*, the B and C class genes are differentially expressed in the male and female flowers early in development, resulting in gender determination [36]. The expression of two B class genes in *S. oleracea* (SpAP3 and SpPI) is only detected throughout the stamen development in the male flowers, confirming the role of B class genes in sex differentiation [35]. The putative AP3 gene of *E. ulmoides* and the SpAP3 gene of *S. oleracea* have c. 70% similarity (Table S2), indicating that the probable role of B class genes in the sex determination process of *E. ulmoides*. The co-occurrence of type II unisexual flowers in both *E. ulmoides* [27] and *S. oleracea* [26], together with the homology of their B class genes suggest a possibility of convergent evolution. Further research on the AP3-like gene in *E. ulmoides* are needed via, for example, transgenic approach or gene editing technique to fully understand its function, which will shed light on our understanding of the hypothesis proposed by Diggle et al. [26].

### 3.3. Segregated SNPs in Sex-Associated Genes in E. ulmoides

In dioecious plants, sex-linked genes are generally polymorphic and segregate between genders [63,64]. SNPs calling from segregated populations of dioecious plant can help to identify the sex-associated SNPs and corresponding loci. For example, at least 12 PAR (pseudoautosomal region) genes were identified in the white campion (*S. latifolia*) using RAD-seq approach [65]. The whole-genome scans of poplar (*Populus balsamifera* and *P. trichocarpa*) discovered that hundreds of SNPs were significantly associated with sex and located in ten different genomic regions [66]. We conducted SNP calling analysis in the 116 DEGs finding that a total of five DEGs were polymorphic with common SNPs in each sex type (Table 3 and Table S4). The B class AP3-like gene (Cluster-47702.80936) was suggested to be polymorphic and segregated along with different sex types (Table S4). We therefore infer that the B class AP3-like gene could be one of the potential candidates of sex determination or sex-associated genes in *E. ulmoides*.

Moreover, another four non-MADS box DEGs (Cluster-47702.79497, Cluster-47702.45188, Cluster-47702.80197 and Cluster-47702.38156) segregated within the six selected individuals according to sex (Table 3 and Table S4). Two (Cluster-47702.79497 and Cluster-47702.45188) of the above four genes were enriched in the KEGG pathways of “ribosome biogenesis in eukaryotes” and “basal transcription factors” respectively (Figure 4 and Table S2). Another one (Cluster-47702.80197) was involved in the hydrolase activity (Figure 4 and Table S2). The function of the rest one (Cluster-47702.38156) was unknown by far (Table S2). The above four genes were also aligned with the reported sex-linked regions of *Carica papaya* [67], *S.* *viminalis* [68], *Fragaria chiloensis* [69], *Mercurialis annua* [70], *S. latifolia* [65], *R.* *hastatulus* [23] and two species of *Populus* [66]. However, no homologous fragments of the putative sex-associated genes between *E. ulmoides* and other species were detected. Distant phylogenetic relationship among *E. ulmoides* and those above species [1] is probably the major reason for none overlap between their gender-specific genes. The evolutionary origin of sex-determination genes in different lineages of flowering plants can be traced when more plant sex-determination sequences are available. It is also possible that polymorphic sex-biased expressed genes link together in the antagonist non-recombining regions i.e., SDR (sex determination regions) to segregate consistently [41,71]. The four DEGs are likely linked with the aforementioned B class MADS box TF gene (AP3-like) in the *E. ulmoides* genome. We tend to believe that those putative sex-associated genes in the leaves detected here might be differently expressed in the reproductive organs as well, e.g., flower buds [72], given that the sex-associated genes probably express constitutively in vegetative and reproductive tissues in dioecious plants [27,37]. However, further studies are needed.

A high level of genetic diversity at the population level of *E. ulmoides* was reported based on SSR (simple sequence repeats) markers (*H*_E_ = 0.716, [73]). Here in this study SNPs were frequently occurred in the expressed genes of *E. ulmoides* (Table 4 and Table S5). In total 119,411 putative SNPs were detected in the gene regions with an average of one SNP per 980 bp, showing more than five folds higher of density than in the rubber tree (*H. brasiliensis*) gene regions (one SNP per 5.2 kb) [74]. The dioecious sexual system of *E. ulmoides* may be one of the factors contributing to the high level of its genetic diversity [73,75]. Furthermore, the plentiful SNP markers detected here could use to construct finer genetic maps of *E. ulmoides* compared to the previously published ones [76,77] to improve the molecular breeding process of *E. ulmoides*. In short, the results obtained in this study provided first insights into the sexual dimorphism of gutta content and the genetic mechanism of sex determination in *E. ulmoides*. These data generated here will facilitate further comprehensive research on the sex differentiation of *E. ulmoides* and the other dioecious plants in the future.

## 4. Materials and Methods

### 4.1. Plant Materials

Three independent full-sib families of *E. ulmoides* growing on the campus of the Northwest A&F University in Yangling, Shanxi, China were selected for this study. In each family one mature male and female individual determined by their distinct male and female flowers in early spring (April) 2016 were sampled. A total of six individuals were sampled with three replicates for each sex. Fresh healthy leaves were collected from each individual before being immersed into liquid nitrogen immediately, and then stored at −80 °C until RNA isolation.

### 4.2. RNA Isolation and Illumina Sequencing

Total RNA was isolated from the leaf samples using a TRIzol Reagent (Invitrogen, Foster City, CA, USA) following the manufacturer’s instructions. The quality of RNA was monitored on the 1% agarose gels and checked using the NanoPhotometer^®^ spectrophotometer (IMPLEN, Westlake Village, CA, USA). The integrity of RNA was further assessed using the RNA Nano 6000 Assay Kit of the Agilent Bioanalyzer 2100 system (Agilent Technologies, Palo Alto, CA, USA). The concentration of the RNA was subsequently measured with a Qubit 2.0 Fluorometer (Life Technologies, Carlsbad, CA, USA).

For each individual a total of c. 1.5 µg RNA was used for subsequent RNA-seq experiment. NEBNext^®^ Ultra^TM^ RNA Library Prep Kit for Illumina^®^ (New England Biolabs, Ipswich, MA, USA) was used to generate one library for each of the examined individuals according to the manufacturer’s instructions. The cDNA fragments with 150–200 bp in length were selectively purified with AMPure XP system (Beckman Coulter, Beverly, MA, USA). Finally, the PCR enriched cDNAs were sequenced on the Illumina HiSeq 2500 platform at Novogene (Beijing, China), to generate PE reads with 150 nucleotides in length.

### 4.3. Transcriptome Assembly and Gene Annotation

Raw reads generated from the RNA-seq were filtered by removing reads containing adapter or ploy-N, and low quality reads to obtain high-quality clean reads. In the meanwhile, the sequencing error rate, Q20, Q30, and GC content of clean data were calculated for assessing the sequencing quality. All the subsequent analyses were based on the clean data.

Since there is no publicly available genome of *E. ulmoides* [6], all the clean reads generated from the six RNA-seq libraries were combined for constructing a reference transcriptome. Trinity [78] was used here for the de novo transcriptome assembly, with min_kmer_cov value set as 2, all the other parameters set as default values. Unigenes were blasted against seven public databases (Nr, Nt, Pfam, KOG, Swiss-Prot, KO and GO) by blastx with e-value < 10^−5^ [79]. The sequence direction and CDS region of unigenes were defined according to the best blast results. For those unigenes that were unable to be aligned to any of the above databases ESTScan [80] was used to predict their CDS and sequence direction.

### 4.4. Gene Differential Expression Analysis

Gene expression level was estimated for each sample by RSEM [81]. Clean reads were mapped back to the assembled unigenes using bowtie2 [82], with mismatch value set as 0. Read counts value for each unigene was normalized by calculating the FPKM value in different samples. To further investigate the sex-biased gene expression patterns, we performed comparative transcriptomic analyses among the pools of samples from the two sex types, with one group including all the males and the other comprising of all the females. Differential expression analyses of these two groups were performed using the DESeq R package v1.10.1 [83]. DESeq provided statistical routines for determining differential expression in digital gene expression data using a model based on the negative binomial distribution [83]. The resulting *p*-values were adjusted using the Benjamini and Hochberg’s approach for controlling the false discovery rate [84]. Genes with *p*_adj_ < 0.05 and |log_2_ (fold change value)| ≥ 1 were considered as DEGs [83], i.e., male- or female-biased genes.

The pheatmap R package (https://cran.r-project.org/web/packages/pheatmap) was applied for K-means cluster analysis [85] to uncover the gene expression patterns for the DEGs in six individuals. The FPKM values of DEGs from each individual were used as input data. Moreover, GO terms [86] enriched in the set of DEGs were implemented by the GOseq R packages based on Wallenius non-central hyper-geometric distribution [87]. WEGO software [88] was employed to plot the distribution of GO classification. KEGG is a database for understanding the high-level functions of the target genes and utilities of the biological system from molecular-level information e.g., large-scale transcriptome or genome data (http://www.genome.jp/kegg/, [89]). We used KOBAS [90] software to test the statistical enrichment of DEGs in KEGG pathways.

### 4.5. MADS Box Genes Analysis

To investigate the sex differentially expressed MADS box genes in *E. ulmoides* iTAK program [91] was used to recognize putative MADS box TFs from the assembled transcriptome. In the meanwhile, the assembled unigenes were analyzed by HMMER v3.1b2 (http://hmmer.org/) to identify differentially expressed MADS box TF genes between the males and females based on the plant TF database (PlantTFDB, http://planttfdb.cbi.pku.edu.cn/).

All the obtained MADS box-like genes were aligned using MUSCLE [92]. An unrooted NJ tree was then constructed with the HKY model in Geneious v9.0 (http://www.geneious.com, [93]), with 1000 bootstrap reps calculation. A putative AP3 gene of *E. ulmoides* differentially expressed in the two sexes was further examined across other 37 angiosperm species. 57 AP3 orthologues in total were collected from *EnsemblPlants* database (http://plants.ensembl.org/) and aligned with the identified AP3-like gene of *E. ulmoides*. *Amborella trichopoda* was defined as outgroup according to the updated APG IV system [1]. Phylogenetic analysis of the alignment was carried out using the ML method in RAxML v.8.2.8 [94] under the model of GTR + CAT. 1000 fast bootstrap ML reps were implemented to assess the relative degree of support (MLBS) for internal nodes. The BI analysis was also performed in MrBayes 3.2.6 [95]. Two runs with four chains were run for 40,000,000 generations under the model of GTR + G, with a sampling of every 1000 generations till convergence (the average standard deviation of split frequencies less than 0.01). After discarding the first 25% of trees as burn-in the remaining trees were used to estimate majority-rule consensus tree with posterior probabilities (PP).

### 4.6. SNPs Calling

Given the sex-associated genes in dioecious plants are polymorphic and segregate along with sex, e.g., in *Actinidia chinensis* [96], *Bryonia dioica* [97], and *Hippophae rhamnoides* [98], we here mapped the clean reads of each male and female individual to the assembled unigenes for SNPs calling to identify potential sex-associated candidates in *E. ulmoides*. Picard-tools v1.41 (http://broadinstitute.github.io/picard/) and samtools v0.1.18 [99] were used to sort and remove duplicated reads and merge the bam alignment results of each sample. GATK3 software [100] was further applied to perform SNPs identification. Raw vcf files were filtered with GATK standard filter method; other parameters were set as follows: cluster, 3; WindowSize, 35; MQRankSum < −12.5, ReadPosRankSum < −8.0, DP < 10, FS > 60.0, SOR > 4.0. 

The frequency and distribution of SNPs occurrence in the expressed genes of *E. ulmoides* were surveyed. Then the SNPs in DEGs i.e., sex-biased expressed genes were examined to investigate their most likely associations with the dioecy of *E. ulmoides*. To obtain a more solid result more stringent cutoff values were applied as follows: (1) at least five unique reads in each individual covered the same nucleotide position; (2) the genes must be polymorphic (in terms of SNPs) in the six individuals; (3) the SNPs must be common in each sex type, i.e., males shared one SNP and females shared the other one. The SNPs that satisfied the above criteria were considered as potential sex-associated SNPs and the corresponding genes were identified as the putative sex-associated genes.

## 5. Conclusions

NGS-based transcriptome sequencing was applied to investigate the genes showing sex-biased expression patterns in the vegetative leaf tissues of *E. ulmoides*, a representative of dioecious woody plant with sex differentiation at initiation. Our results revealed 116 DEGs between the male and female leaves. A number of candidate genes related to the sexual dimorphism of gutta yield in the leaves were characterized. Five putative sex-associated genes including a MADS box TF gene involved in the male organ identity were identified. High frequency of SNPs was also detected in the expressed genes. Collectively, this study will shed light on our understanding of the genetic regulation of sex expression in *E. ulmoides* and will also facilitate further comprehensive research on more dioecious species in the future.

## Figures and Tables

**Table 1:** Statistics of RNA-seq.

Sample/Item *	Raw Reads	Clean Reads	Size of Clean Data (G)	Error (%)	Q20 (%)	Q30 (%)	GC Content (%)
EUCO_M1	52,628,596	50,575,362	7.59	0.02	95.55	89.51	45.18
EUCO_M2	55,644,868	53,387,028	8.01	0.02	95.44	89.63	40.91
EUCO_M3	60,088,910	57,307,276	8.60	0.02	95.04	89.45	40.81
EUCO_F1	52,436,122	49,456,850	7.42	0.02	95.49	89.50	43.20
EUCO_F2	53,027,850	50,069,058	7.51	0.02	95.47	89.59	41.10
EUCO_F3	53,303,500	50,531,298	7.58	0.02	95.46	89.75	40.56
Total	327,129,846	311,326,872	46.71	/	/	/	/

* Abbreviation: EUCO_M, *Eucommia ulmoides* males; EUCO_F, *Eucommia ulmoides* females. The numbers 1/2/3 represent different individuals.

**Table 2:** Summary of de novo transcriptome assembly.

Item	Values
Number of contigs	289,704
Minimum length of contigs (bp)	201
Mean length of contigs (bp)	540
Max length of contigs (bp)	12,536
N50 of contigs (bp)	705
Total length of contigs (bp)	156,318,751
Total number of unigenes	148,595
Average sequence size of unigenes (bp)	801
Length of all unigenes (bp)	117,298,413
N50 of unigenes (bp)	1064

**Table 3:** Putative sex-associated genes in *Eucommia ulmoides*.

Gene_ID	Position *	EUCO_F1 *	EUCO_F2	EUCO_F3	EUCO_M1 *	EUCO_M2	EUCO_M3	Regulation
Cluster-47702.80936	1101	C	C	C	G	G	G	Male-biased expression
Cluster-47702.80197	360	T	T	T	C	C	C	Male-biased expression
	460	G	G	G	A	A	A	
Cluster-47702.38156	538	T	T	T	C	C	C	Male-biased expression
	596	G	G	G	T	T	T	
Cluster-47702.79497	297	A	A	A	G	G	G	Male-biased expression
	312	C	C	C	T	T	T	
Cluster-47702.45188	947	T	T	T	G	G	G	Male-biased expression
	968	T	T	T	C	C	C	

* Abbreviation: EUCO_M, *Eucommia ulmoides* males; EUCO_F, *Eucommia ulmoides* females. The numbers 1/2/3 represent different individuals. * Position was calculated started from the 5′ end to the 3′ end.

**Table 4:** Density and distribution of SNPs (single nucleotide polymorphisms) in *Eucommia ulmoides* genes derived from the assembled transcriptome.

Type	Count	Occurrence per kb
Transition		
C/T	38,758	0.33
A/G	39,805	0.34
Transversion		
A/T	11,838	0.10
A/C	10,256	0.09
T/G	10,298	0.09
C/G	8456	0.07
Total	119,411	1.02
SNP position in codon		
First	22,235	
Second	8889	
Third	22,148	

## Supplementary Materials

Supplementary materials are available online. Table S1: Annotation information of non-redundant consensus sequences of *Eucommia ulmoides*. Table S2: The functional annotation and enrichment analysis of DEGs (differentially expressed genes) between the males and the females of *Eucommia ulmoides*. Table S3: The putative MADS box TF (transcription factor) genes in *Eucommia ulmoides*. Table S4: The SNPs (single nucleotide polymorphisms) calling in DEGs (differentially expressed genes) between the males and the females of *Eucommia ulmoides*. Table S5: SNPs (single nucleotide polymorphisms) occurrence in the expressed genes in each *Eucommia ulmoides* individual. Figure S1: The pathway of pyruvate metabolism. Two unigenes of *Eucommia ulmoides* were assigned to the pyruvate metabolism by KEGG enrichment analysis. The gene/protein name in a red and green box represents male- and female-biased genes, respectively.
