# Supplementary material for: Identification of the Sex-Biased Gene Expression and Putative Sex-Associated Genes in Eucommia ulmoides Oliver Using Comparative Transcriptome Analyses
Source: Molecules. 2017 Dec 18;22(12):2255. doi: 10.3390/molecules22122255 (PMC6149867; doi:10.3390/molecules22122255)
Supplement: Supplementary file 1 [file molecules-22-02255-s001.zip › Supplementary Materials-for proof/Table S1-revision.docx]

**Table S1. Annotation information of non-redundant consensus sequences of *Eucommia ulmoides*.**

| **Database^*^** | **Number of Unigenes** | **Percentage (%)** |
| --- | --- | --- |
| Annotated in NR | 71,810 | 48.32 |
| Annotated in NT | 49,521 | 33.32 |
| Annotated in KO | 31,746 | 21.36 |
| Annotated in SwissProt | 57,573 | 38.74 |
| Annotated in PFAM | 54,515 | 36.68 |
| Annotated in GO | 55,473 | 37.33 |
| Annotated in KOG | 24,028 | 16.17 |
| Annotated in all databases | 12,799 | 8.61 |
| Annotated in at least one database | 84,271 | 56.71 |
| Total unigenes | 148,595 | 100 |

**^*^** NR (NCBI non-redundant protein sequences); NT (NCBI non-redundant nucleotide sequences); PFAM (Protein family); KOG (euKaryotic Ortholog Groups); Swiss-Prot (A manually annotated and reviewed protein sequence database); KO (KEGG Ortholog); GO (Gene Ontology).
