# Supplementary material for: Identification of the Sex-Biased Gene Expression and Putative Sex-Associated Genes in Eucommia ulmoides Oliver Using Comparative Transcriptome Analyses
Source: Molecules. 2017 Dec 18;22(12):2255. doi: 10.3390/molecules22122255 (PMC6149867; doi:10.3390/molecules22122255)
Supplement: Supplementary file 1 [file molecules-22-02255-s001.zip › Supplementary Materials-for proof/Table S5.docx]

**Table S5. SNPs (single nucleotide polymorphisms) occurrence in the expressed genes in each *Eucommia ulmoides* individual.**

| **Sample^*^** | **Total SNPs** | **Non-coding SNPs** | **Coding SNPs** | **Synonymous SNPs** | **Nonsynonymous SNPs** |
| --- | --- | --- | --- | --- | --- |
| EUCO_F1 | 54,286 | 29,255 (53.89%) | 25,031 (46.11%) | 15,976 (29.43%) | 9,055 (16.68%) |
| EUCO_F2 | 52,177 | 29,028 (55.63%) | 23,149 (44.37%) | 14,719 (28.21%) | 8,430 (16.16%) |
| EUCO_F3 | 52,234 | 28,156 (53.90%) | 24,078 (46.10%) | 15,771 (30.19%) | 8,307 (15.90%) |
| EUCO_M1 | 32,570 | 16,868 (51.79%) | 15,702 (48.21%) | 10,262 (31.51%) | 6,606 (20.28%) |
| EUCO_M2 | 48,037 | 26,318 (54.79%) | 21,719 (45.21%) | 13,365 (27.82%) | 8,354 (17.39%) |
| EUCO_M3 | 57,340 | 30,663 (53.48%) | 26,677 (46.52%) | 17,146 (29.90%) | 9,531 (16.62%) |

^*^ EUCO_M: Males; EUCO_F: Females, the numbers 1/2/3 represent different individuals.
