# Supplementary figures and images for: Identification of the Sex-Biased Gene Expression and Putative Sex-Associated Genes in Eucommia ulmoides Oliver Using Comparative Transcriptome Analyses
Source: Molecules. 2017 Dec 18;22(12):2255. doi: 10.3390/molecules22122255 (PMC6149867; doi:10.3390/molecules22122255)

# PYRUVATE METABOLISM

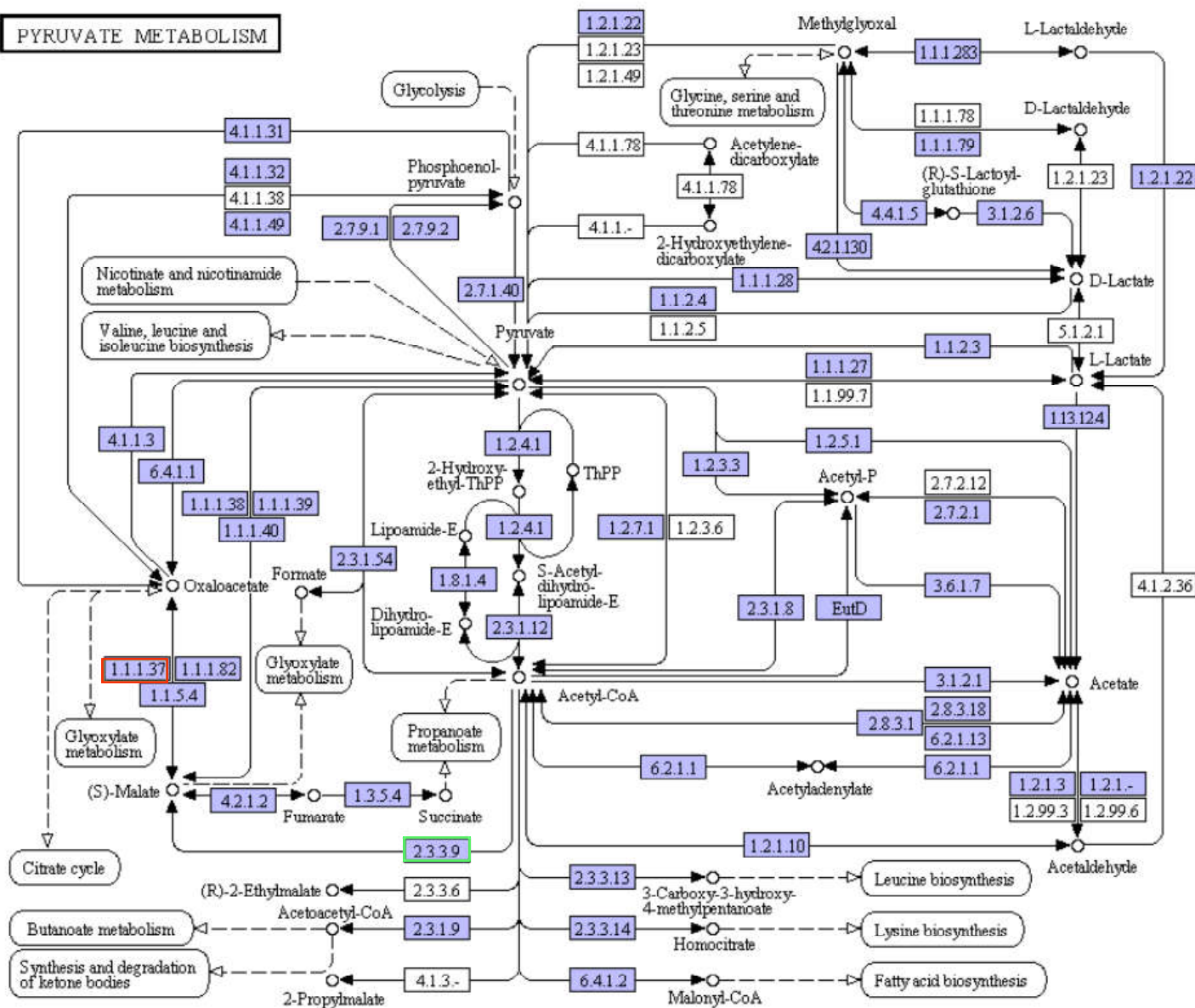

Supplement: Supplementary file 1 [file molecules-22-02255-s001.zip › Supplementary Materials-for proof/Figure S1.pdf]
